# Calcium-deficient Hydroxyapatite as a Potential Sorbent for Strontium

**DOI:** 10.1038/s41598-017-02269-z

**Published:** 2017-05-18

**Authors:** Yurina Sekine, Ryuhei Motokawa, Naofumi Kozai, Toshihiko Ohnuki, Daiju Matsumura, Takuya Tsuji, Riku Kawasaki, Kazunari Akiyoshi

**Affiliations:** 1 Materials Sciences Research Center, Japan Atomic Energy Agency, 2-4 Shirakata-Shirane, Naka-gun, Tokai, Irabaki 319-1195 Japan; 2 Advanced Science Research Center, Japan Atomic Energy Agency, 2-4 Shirakata-Shirane, Naka-gun, Tokai, Ibaraki 319-1195 Japan; 3 Laboratory for Advanced Nuclear Energy, Institute of Innovative Research, Tokyo Institute of Technology, Ookayama 2-12-1, Meguro, Tokyo 152-8550 Japan; 4 Department of Polymer Chemistry, Graduate School of Engineering, Kyoto University, Katsura, Nishikyo-ku, Kyoto 615-8510 Japan; 5 ERATO Bio-nanotransporter Project, Japan Science and Technology Agency, Kyoto University, Kastura, Nishikyo-ku, Kyoto 615-8510 Japan

## Abstract

A calcium (Ca)-deficient hydroxyapatite was investigated for its potential to remove Sr^2+^ from environmentally relevant water. We conducted sorption tests on solutions containing magnesium ion (Mg^2+^) and calcium ion (Ca^2+^) as competing cations at a strontium ion (Sr^2+^) concentration of 0.05 mmol/L. The Ca-deficient hydroxyapatite maintained a high Sr^2+^ sorption ratio of above 80% in the presence of Mg^2+^ and Ca^2+^ at the concentrations between 0.1 and 1.0 mmol/L, whereas the stoichiometric hydroxyapatite showed a lower ratio even in the presence of small amounts of Mg^2+^ and Ca^2+^ (72% for Mg^2+^ and 51% for Ca^2+^ at 0.1 mmol/L). For solutions with various Sr^2+^ concentrations between 0.01 and 10 mmol/L, Ca-deficient hydroxyapatite exhibited a higher Sr^2+^ sorption ratio than stoichiometric hydroxyapatite. The bonding states of Sr^2+^ on the Ca-deficient hydroxyapatite were evaluated by extended X-ray absorption fine structure measurements. The results indicated that there are specific sorption sites in Ca-deficient hydroxyapatite where Sr^2+^ is stably and preferentially immobilized.

## Introduction

Since the accident at the Fukushima-Daiichi nuclear power plant (FNPP) in 2011, there has been an immense need for novel ways to remove radioisotopes from contaminated water. A substantial number of radioisotopes such as strontium-90 (^90^Sr), cesium-134 (^134^Cs), and cesium-137 (^137^Cs) were released from FNPP into the environment, thereby contaminating the seawater, groundwater, and land surface^1–3^. The radioactivity of the Sr and Cs released from FNPP onto the land surface was estimated to be around 8.56 PBq for ^90^Sr and 288 PBq for ^134^Cs and ^137^Cs, whereas that of the strontium and cesium directly released into the ocean was estimated to be 52 GBq for ^90^Sr and 3.5 PBq for ^134^Cs and ^137^Cs^4, 5^. Of these radioisotopes, ^90^Sr poses a major threat to human health and the environment because it can replace calcium (Ca) in bones and in plants, leading to genetic mutations^6^. Thus, studying the migration of ^90^Sr in solution and determining suitable decontamination procedures are important issues to be researched.

Sorbent materials such as minerals^5, 7–13^ and polymeric materials^13^ have been used for the removal of ^90^Sr so far, however, development of a sorbent with both high sorption capacity and high ion selectivity is essential. Usually, several types of ion species such as magnesium ion (Mg^2+^) and calcium ion (Ca^2+^) are present in contaminated seawater and groundwater^5, 14, 15^, making it difficult for sorbents to maintain their targeted ion sorption capacity. Sorbents with Cs^+^ selectivity have been developed^16, 17^, but materials with Sr selectivity have hardly been reported.

Hydroxyapatites (HAPs), which are calcium phosphate crystals, are the main components of our teeth and bones. They are of considerable interest in environmental and biomedical fields due to their ion-exchange ability and sorption capacity^5, 12, 18, 19^. In HAP crystals, Ca^2+^ ions are located at two distinct crystallographic sites, which provide exchange sites for a wide range of divalent cations^20–22^. As Sr^2+^ and Ca^2+^ have the same charge and a similar ionic radii (1.2 Å^23^ and 1.0 Å^23^, respectively), Sr^2+^ can be adsorbed onto HAP with high efficiency^4, 12, 24^. This preferential selectivity for Sr would make HAP an effective absorbent for the removal of Sr^2+^. In fact, a HAP reactive barrier has already been successfully trialed at the Hanford nuclear site in the USA to prevent the migration of ^90^Sr into the Columbia River^25^. Recently, HAPs are also being tested as a potential remediation material at the FNPP site^15^.

The chemical composition of HAPs can be modified from the stoichiometric form (Ca_10_(PO_4_)_6_(OH)_2_) to the Ca-deficient form (Ca_10−x_(HPO_4_)_x_(PO_4_)_6−x_(OH)_2−x_) by selecting appropriate Ca/P molar ratios^18, 26–28^. Natural HAPs in teeth and bones also have a nonstoichiometric form^18^. Moreover, HAPs can maintain their crystal structure regardless of the Ca/P molar ratio; thus, Ca-deficient HAP has vacant Ca^2+^ sites within its crystal structure^18, 27, 28^. It is known that the targeted ion-sorption properties of HAPs strongly depend on the Ca/P molar ratio^29–32^. In this study, we investigated the potential of Ca-deficient HAP for cleaning up contaminated water via Sr^2+^ sorption.

## Results

### Material characterization

**Figure 1:**
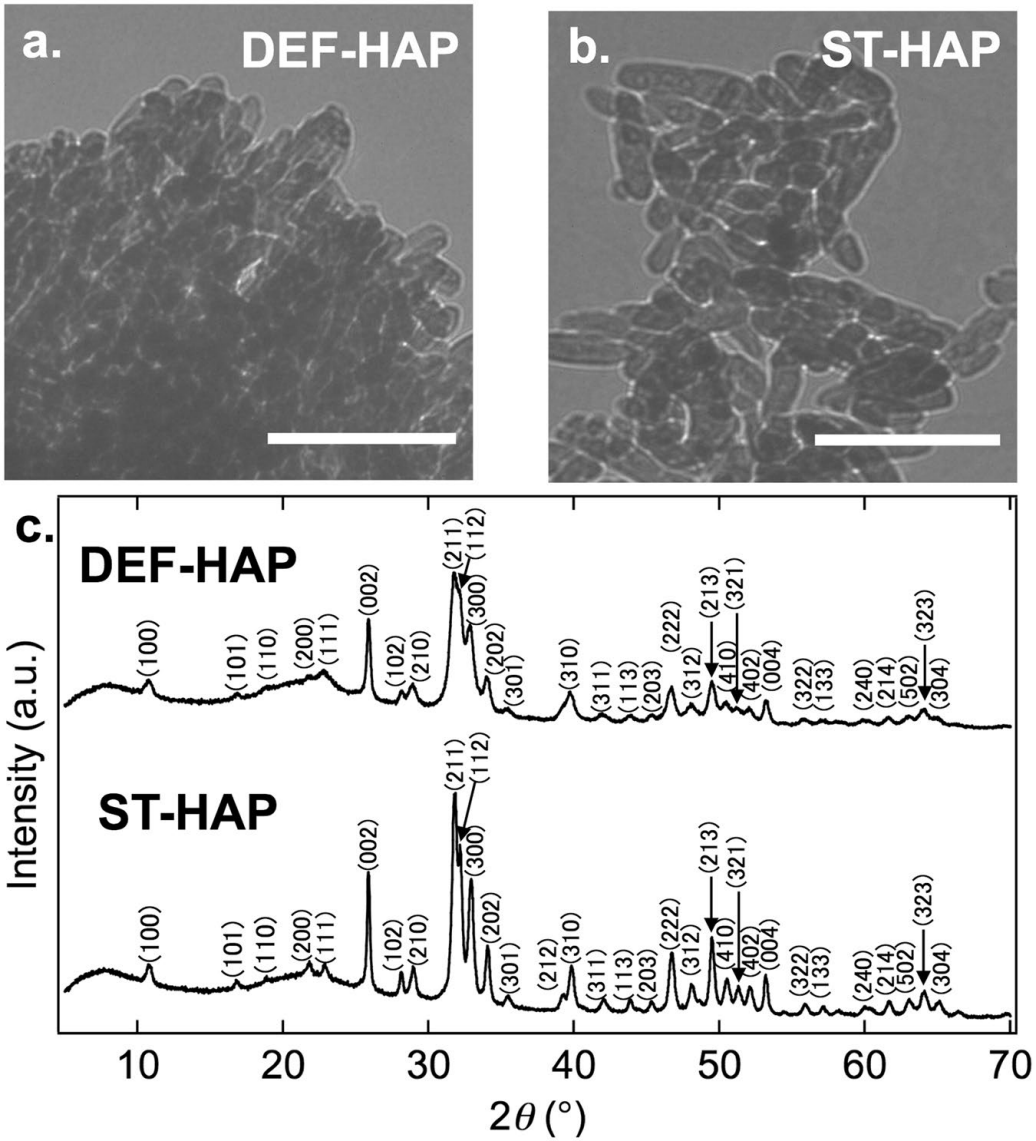
HAP crystal structures. Panels (a,b) show the TEM images of DEF-HAP and ST-HAP, respectively. The scale bars represent 200 nm. (**c**) X-ray powder diffraction profiles of DEF-HAP and ST-HAP. Miller indices corresponding to an hydroxyapatite phase ((*P*6_3_/*m*)^24^ are indicated.

The physical–chemical characterization of Ca-deficient hydroxyapatite (DEF-HAP, Ca/P = 1.38) and stoichiometric hydroxyapatite (ST-HAP, Ca/P = 1.68) is summarized in Table 1. Transmission electron microscopy (TEM) images of the materials (Fig. 1(a) and (b)) show that both DEF-HAP and ST-HAP form rod-like crystals and that the crystals of DEF-HAP are smaller than those of ST-HAP. The size of the crystals was estimated by analyzing these images; the average length (*L*) of the crystals of DEF-HAP (43 ± 5.7 nm) was approximately 46 nm shorter than that of ST-HAP (89 ± 17 nm), whereas their average widths (*W*) were almost identical (~17 nm) (Table 1). The X-ray powder diffraction (XRD) profiles of DEF-HAP and ST-HAP (Fig. 1(c)) have peaks in the 2*θ* range of 10–70°, and the positions of the peaks are similar to those of hydroxyapatites having a hexagonal structure (*P*6_3_/*m*)^24^. The broader peak patterns observed for DEF-HAP might be due to its smaller crystal sizes, which agrees with the TEM observations. A broad band observed at a 2*θ* angle in the range 15°–28° might have originated from an amorphous component in the HAPs. Notably, the integrated intensities of the broad bands for DEF-HAP and ST-HAP are almost identical. At pH 7, the zeta potentials of DEF-HAP and ST-HAP were −20.1 and −5.46 mV, respectively.

**Table 1:** Physical–chemical characterization of DEF-HAP and ST-HAP.

	Ca/P ratio	Crystal size (nm)		Zeta potential (mV) at pH 7
		*L* ^a^	*W* ^b^	
DEF-HAP	1.38	43 ± 5.7 nm	17 ± 2.8 nm	−20.1
ST-HAP	1.68	89 ± 17 nm	17 ± 6.9 nm	−5.46

^a^average length of the crystals.

^b^average width of the crystals.

### Effect of competing cations

The effect of competing cations on the Sr^2+^ sorption capacity of DEF-HAP and ST-HAP was evaluated using an aqueous solution containing HAPs (35 mg), Sr^2+^ (0.05 mmol/L), and either Mg^2+^ or Ca^2+^ (0–50 mmol/L) in MilliQ water (7 mL). The solution was stirred for 5 h at pH 7. The supernatant was obtained by centrifugation and filtration, and the concentration of Sr^2+^ was determined using inductively coupled plasma-mass spectrometry (ICP-MS). Notably, the equilibrium time for the sorption was confirmed to be 1 h; hence, five hours were sufficient to evaluate the sorption properties of the HAP materials (refer to Fig. S1 in the Supplementary Information). Based on the concentration of Sr^2+^ in the supernatant, the sorption efficiency (*A*_*eff*_) of DEF-HAP and ST-HAP was calculated as follows:

1 $$\,{A}_{eff}( \% )=100\times (1-\frac{C}{{C}_{0}}),$$

where *C*_*0*_ and *C* are the initial and final (after 5 h) concentrations (mg/L) of Sr^2+^, respectively.

**Figure 2:**
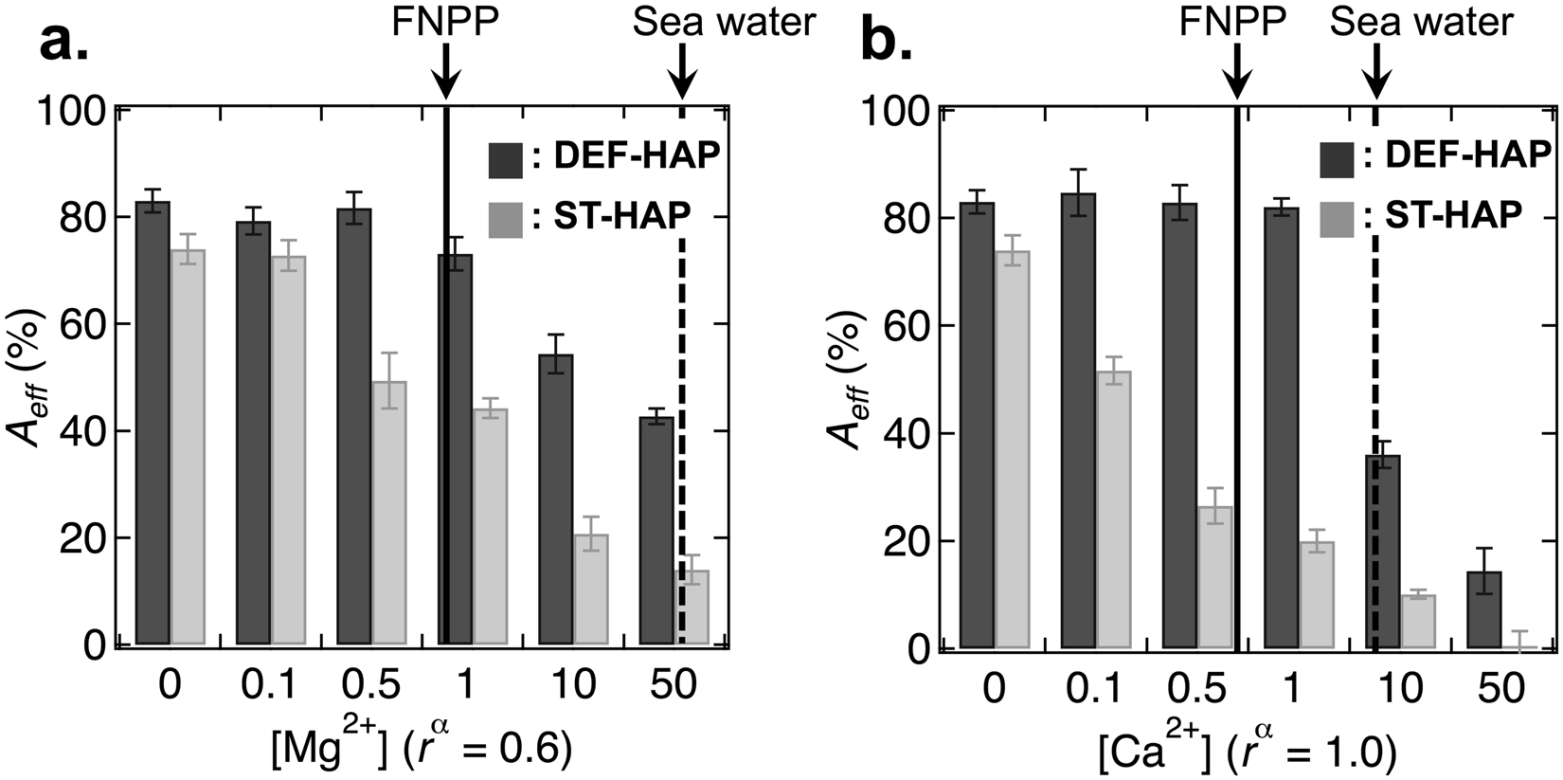
Effect of competing ions on the Sr^2+^ sorption capacity of HAPs. The change in *A*_*eff*_ for DEF-HAP (dark gray) and ST-HAP (light gray) as a function of the concentration (mmol/L) of (**a**) Mg^2+^ ([Mg^2+^]) and (**b**) Ca^2+^ ([Ca^2+^]) is shown. The dashed and solid lines represent the approximate average [Mg^2+^] and [Ca^2+^] in seawater^14^ (52 mmol/L for Mg^2+^ and 9.4 mmol/L for Ca^2+^) and in FNPP ground water^15^ (0.86 mmol/L for Mg^2+^ and 0.70 mmol/L for Ca^2+^), respectively. The hydrated ionic radii of Mg^2+^ and Ca^2+^ are 0.6 Å and 1.0 Å, respectively. *α* is the ionic hydrated radius (Å).

As shown in Fig. 2(a), DEF-HAP demonstrates a high *A*_*eff*_ in the absence of Mg^2+^ and Ca^2+^ (83%), and the values remain almost constant in the presence of Mg^2+^ and Ca^2+^ at concentrations between 0.1 and 1.0 mmol/L. The values decrease as the concentration increases above 1.0 mmol/L, decreasing to 42% for Mg^2+^ and 14% for Ca^2+^ at 50 mmol/L. Conversely, ST-HAP exhibits a lower *A*_*eff*_ than DEF-HAP in the absence of Mg^2+^ and Ca^2+^ (74%) and the values decrease even in the presence of small amounts of Mg^2+^ and Ca^2+^ (72% for Mg^2+^ and 51% for Ca^2+^ at 0.1 mmol/L). These values significantly decrease to 14% for Mg^2+^ and 0.5% for Ca^2+^ at 50 mmol/L. The dashed and solid lines in Fig. 2(a) and (b) denote the Mg^2+^ and Ca^2+^ concentrations in seawater and groundwater at FNPP, respectively. Our results demonstrate that DEF-HAP can maintain its Sr^2+^ sorption capacity under the existing groundwater conditions at FNPP.

### Dependence on initial concentration

**Figure 3:**
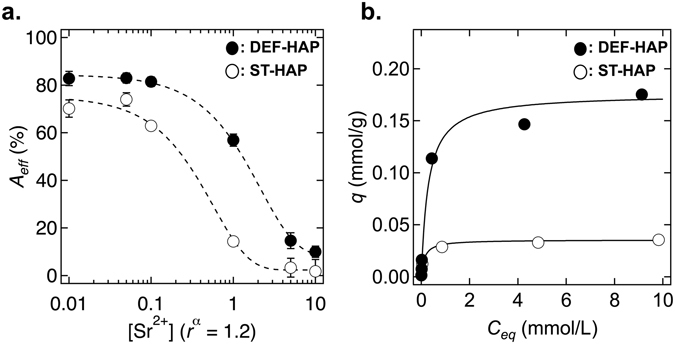
Dependence of initial concentration of Sr^2+^ on the sorption capacity of HAPs. (**a**) *A*_*eff*_ of DEF-HAP (closed circles) and ST-HAP (open circles) as a function of Sr^2+^ concentration ([Sr^2+^]) between 0.01 and 10 mmol/L. The dashed lines are guides to the eye. The hydrated ionic radius of Sr^2+^ is 1.2 Å. *α* is the ionic hydrated radius (Å). (**b**) Sorption isotherm of DEF-HAP (closed circles) and ST-HAP (open circles) fitted to a Langmuir isotherm model using the values of *q*_*max*_ and *b* (solid lines) (refer to Fig. S2 in the Supplementary Information).

We investigated the dependence of the Sr^2+^ concentration on the sorption capacity of DEF-HAP and ST-HAP using aqueous solutions initially containing 0.01, 0.05, 0.1, 1, 5, and 10 mmol/L of Sr^2+^ at pH 7. Their supernatants were obtained after 5 h, and *A*_*eff*_ was estimated using Eq. (1). As shown in Fig. 3(a), the *A*_*eff*_ of DEF-HAP is 82% at 0.01 mmol/L; this value remains almost constant as the concentration is increased to 0.1 mmol/L. For higher Sr^2+^ concentrations, *A*_*eff*_ decreases with an increase in the concentration and decreasing to 10% at 10 mmol/L. For ST-HAP, the *A*_*eff*_ at 0.01 mmol/L is 70%, which significantly decreases above 0.05 mmol/L. The values of *A*_*eff*_ for DEF-HAP are higher than those of ST-HAP at all measured concentrations. The most significant difference was observed at 1 mmol/L where the value of *A*_*eff*_ for DEF-HAP was 60%, which is 75% higher than that of ST-HAP at the same concentration, that is, 15%.

Figure 3(b) shows the sorption isotherms of DEF-HAP and ST-HAP fitted to a Langmuir isotherm model^33^. The model assumes monolayer coverage on a surface with a finite number of identical sites and is represented as follows^33^:

2 $$\frac{{C}_{e}}{{q}_{e}}=\frac{1}{{q}_{{ma}{{x}}^{b}}}+\frac{{C}_{e}}{{q}_{\max }},$$

where *C*_*e*_ is the equilibrium concentration (mg/L) of Sr^2+^, *q*_*e*_ is the amount of adsorbed Sr^2+^ at equilibrium (mg/g), and *q*_*max*_ (mg/g; maximum sorption capacity) and *b* (affinity) are Langmuir constants, respectively. The parameters *q*_*max*_ and *b*, evaluated via Eq. (2) (refer to Fig. S2 in the Supplementary Information), and the coefficients of determination (*R* ^*2*^) are summarized in Table 2. The result shows that DEF-HAP (15.4 mg/g) has a larger maximum sorption capacity for Sr^2+^ than ST-HAP (3.11 mg/g).

**Table 2:** Langmuir isotherm parameters for DEF-HAP and ST-HAP.

	*R* ^*2*^	*q*_*max*_ (mg/g)	*b*
HAP400	0.987	15.4	3.50
HAP100	0.981	3.11	7.70

### Extended X-ray absorption fine structure (EXAFS) measurements

**Figure 4:**
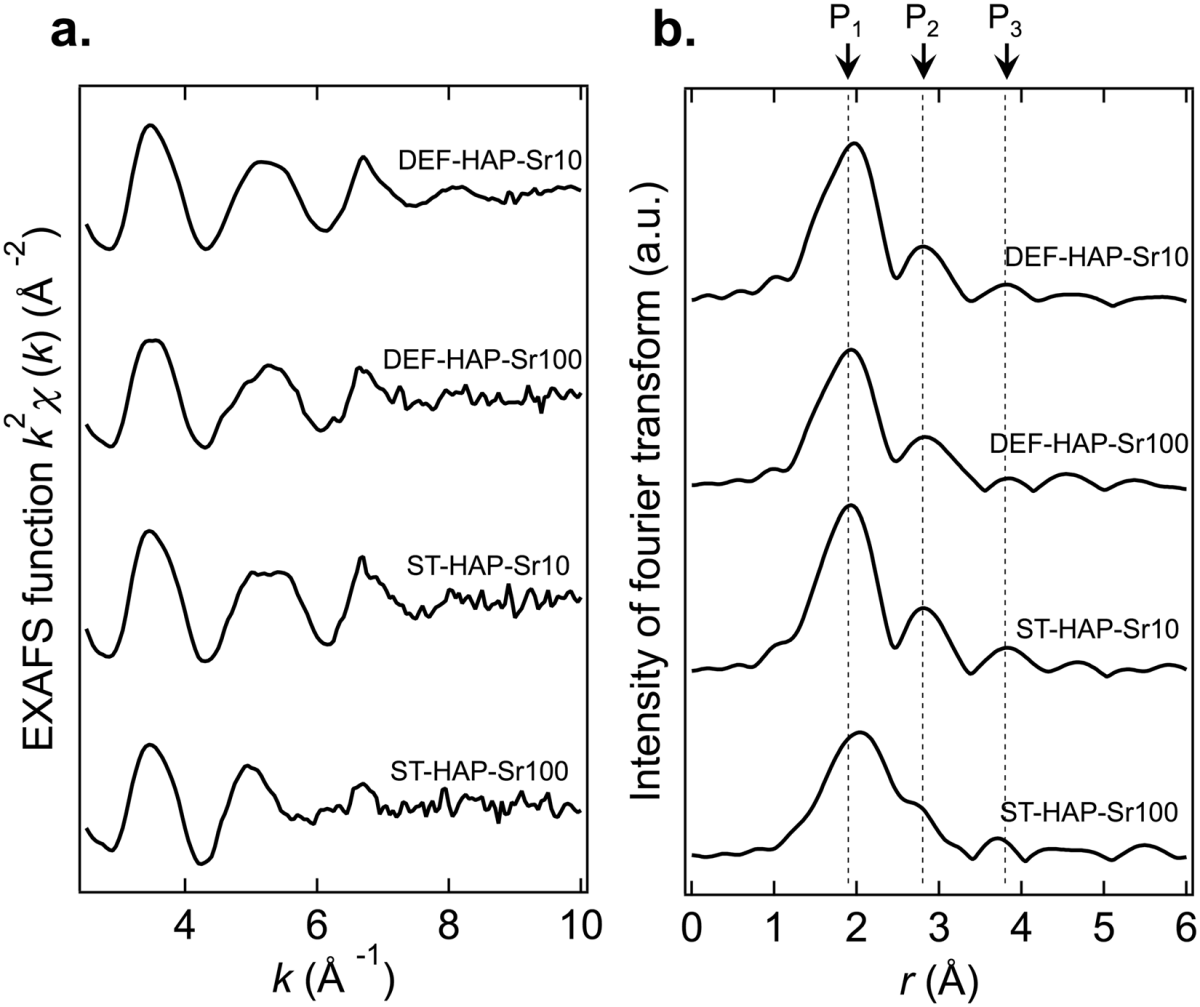
Sr *K*-edge EXAFS analysis of the HAP samples. (**a**) EXAFS spectra of DEF-HAP and ST-HAP exposed to 10 and 100 mmol/L of Sr^2+^, where *k* is the wavenumber. (**b**) Fourier transforms of the EXAFS spectra where *r* is the interatomic distance. Notably, the *r* does not take phase shifts into account. Transforms were conducted with *k* ^2^ weighting. Three peaks (P_1_, P_2_, and P_3_) are observed at around *r* = 1.9, 2.8, and 3.8 Å in all samples.

The Sr^2+^ sorption mechanism on DEF-HAP and ST-HAP was investigated via EXAFS measurements. Figure 4(a) and (b) shows the EXAFS spectra of DEF-HAP and ST-HAP around the *K*-edge of Sr and the Fourier transforms of DEF-HAP and ST-HAP at concentrations of 10 and 100 mmol/L of Sr^2+^ (these samples are referred to as DEF-HAP-Sr10, DEF-HAP-Sr100, ST-HAP-Sr10, and ST-HAP-Sr100). The features of the spectra are similar to those of a previously reported Sr-doped hydroxyapatite^22^. As shown in Fig. 4(b), three peaks (P_1_, P_2_, and P_3_) are observed around *r* (interatomic distance) = 1.9, 2.8, and 3.8 Å for all samples. These peaks are attributed to two O shells and one P shell nearest to the Sr^22, 34, 35^. The EXAFS spectrum obtained for ST-HAP-Sr100 differs from that of the other samples as the P_1_ peak shifts to a longer *r*, whereas P_2_ shifts to a shorter *r* when ST-HAP is exposed to 100 mmol/L of Sr^2+^; moreover, the peak intensity of P_3_ decreases. The spectral line shapes of DEF-HAP are similar irrespective of the Sr^2+^ concentration.

## Discussion

Sorption tests for HAPs in the presence of competing ions showed that the *A*_*eff*_ of both DEF-HAP and ST-HAP is affected less by Mg^2+^ than by Ca^2+^ (Fig. 2). Divalent cations can be exchanged with Ca^2+^ in crystals; moreover, ions with hydrated radii in the range of 0.9–1.3 Å are easily exchanged as compared with other ions^36^. Since the hydrated ionic radius of Sr^2+^ is 1.2 Å^23^, sorption of Sr^2+^ might occur prior to that of Mg^2+^, which has a hydrated ionic radius of 0.6 Å^23^. With Ca^2+^ as the competing ion, DEF-HAP maintains its *A*_*eff*_ for a specific range of concentrations. This means the Sr^2+^ sorption capacity of DEF-HAP is equal to or greater than its Ca^2+^ sorption capacity. The result implies that the vacant Ca^2+^ sites in DEF-HAP are slightly larger than the ionic radius of Ca^2+^, making them more suitable for Sr^2+^ than for Ca^2+^.

The *A*_*eff*_ of DEF-HAP is higher than that of ST-HAP at all the tested concentrations, as shown in Fig. 3. The sorption of divalent cations onto HAPs occurs both at the surface and within the crystals^21^. In aqueous solutions, strontium mainly exists as Sr^2+^ at pH 7^37^, and these positively charged species are actively adsorbed at the negatively charged sites in the HAPs by electrostatic attraction. Thus, the higher negative charge on the surface of DEF-HAP (zeta potential of −20.1 mV) compared with that on the surface of ST-HAP (zeta potential of −5.46 mV) contributes to its higher absorptivity of Sr^2+^. The higher negative zeta potential of DEF-HAP might be due to the existence of PO_4_^3−^, OH^−^, or HPO_4_^−^ surface ions; similar observations have also been made for other Ca-deficient hydroxyapatites^38, 39^. These results suggest that DEF-HAP has a higher Sr^2+^-selective sorption capacity owing to its negatively charged surface and the vacant Ca^2+^ sites within its crystal structure.

In Fig. 4(b), EXAFS spectra of DEF-HAP for different Sr^2+^ concentrations appear to be independent of concentration, suggesting that the sorption sites of DEF-HAP remain unchanged for all the studied conditions; however, the ST-HAP spectra vary with the Sr^2+^ concentration. In the spectrum of ST-HAP-Sr100, P_1_, which is related to the O shell nearest to the Sr, shifts to a larger *r*, indicating that there are two types of sites providing shorter and longer Sr–O distances in ST-HAP. This implies that one site providing a short Sr–O distance is occupied at a certain Sr^2+^ concentration below 100 mmol/L; moreover, the other site providing long Sr–O distance becomes more dominant in ST-HAP. The spectra for DEF-HAP suggest that DEF-HAP only contains sites that provide a shorter Sr–O distance, in which O strongly interacts with Sr^2+^. This might contribute to the higher value of *q*_*max*_ observed in Fig. 3.

In conclusion, Sr^2+^ sorption tests in the presence of competing cations demonstrate that DEF-HAP has a higher Sr^2+^ sorption selectivity than ST-HAP even for conditions similar to those in the groundwater at FNPP. The difference in sorptivity for the competing cations indicates that the ionic radius is one of the important factors affecting the sorption of ions onto HAPs and that HAPs are efficient sorbents for Sr^2+^, which has a hydrated ionic radius of 1.2 Å. Furthermore, DEF-HAP could maintain its Sr^2+^ sorption capacity even in the presence of Ca^2+^. Compared with ST-HAP, DEF-HAP showed a higher *A*_*eff*_ for solutions with various Sr^2+^ concentrations. The EXAFS results demonstrate that compared with ST-HAP, DEF-HAP has significantly more sorption sites where Sr^2+^ can be stably and preferentially sorbed. These results provide useful insights that may assist the future development of HAP-based sorption materials for ^90^Sr by controlling their chemical composition.

## Methods

### Materials

Stoichiometric hydroxyapatite (Ca_10_(PO_4_)_6_(OH)_2_, Ca/P = 1.68, ST-HAP) and Ca-deficient hydroxyapatite (Ca_10−x_(HPO_4_)_x_(PO_4_)_6−x_(OH)_2−x_, Ca/P = 1.38, DEF-HAP) were supplied by Taihei Chemical Industrial Co., Ltd., Japan. ST-HAP was synthesized by mixing calcium hydroxide and phosphoric acid solutions^40, 41^. The precipitate was dried at 100 °C. DEF-HAP was synthesized by mixing slurries of calcium hydrogen phosphate dihydrate with an aqueous solution of sodium hydroxide^31, 41^. The precipitate was dried at 150 °C. Strontium chloride (SrCl_2_), magnesium chloride (MgCl_2_), and calcium chloride (CaCl_2_) were purchased from Wako Pure Chemical Industries Co., Ltd., Japan. Sr standard solutions (1 mg/mL) for ICP-MS (PerkinElmer Japan Co., Ltd., United States) were also purchased from Wako Pure Chemical Industries. All materials were used without further purification.

### Characterizations

XRD measurements were performed using an X-ray diffractometer (Ultima IV, Rigaku Co., Ltd. Japan) with Cu-*K*_α_ radiation (λ = 0.15418 nm) at room temperature. The sample was mounted on a glass plate and optically centered on the diffractometer. The diffraction data was collected in steps of 0.02° in for 2*θ* angles between 10° and 70°. TEM images of the HAP samples were obtained using a JEOL TEM (HT-7700, Hitachi, Tokyo, Japan) operated at an electron beam accelerating voltage of 100 kV. The zeta potentials of the HAP samples were measured at room temperature using a Malvern Zetasizer (Marvern Instruments Ltd., Worcestershire, UK). The unit automatically calculates the electrophoretic mobility of the particle and converts the electrophoretic mobility into a zeta potential using the Smoluchowski equation. One milligram of each HAP sample was dispersed in 1 mL of MilliQ water at pH 7 and stirred for 5 h. Each data point in the *A*_*eff*_ plots is an average of approximately three measurements. The pH of the suspension was adjusted using an aqueous solution of 0.1 mmol/L hydrochloric acid (HCl). The pH of the suspension was measured using a digital pH meter (Mettler Toledo Co., Ltd., Switzerland).

### Sorption tests

The effect of competing ions on the Sr^2+^ sorption capacities of DEF-HAP and ST-HAP was investigated using SrCl_2_, CaCl_2_, and MgCl_2_ solutions. We stirred 7 mL of aqueous solutions containing 35 mg of DEF-HAP or ST-HAP and Sr^2+^ (0.05 mmol/L) in MilliQ water for 5 h at room temperature in the presence of Ca^2+^ or Mg^2+^ at concentrations of 0.1, 0, 1, 10, or 50 mmol/L. The initial pH was adjusted to 7 using 0.1 mmol/L of HCl. Aliquots (1 mL) of the suspension were taken after 5 h and were centrifuged at 10,000 g for 5 min. The pH was maintained at 7 for this study since the groundwater at FNPP generally has a pH of 7. The supernatant was filtered through a 0.1-mm polyvinylidene difluoride (PVDF) filter (Millex-GV, Millipore Co., United States). The concentrations of Sr^2+^ in the supernatant were measured via ICP-MS (PerkinElmer Japan Co., Ltd., Japan).

To investigate the dependence of Sr^2+^ concentration on the sorption efficiency, 35 mg of DEF-HAP or ST-HAP was mixed with Sr^2+^ at concentrations of 0.01, 0.05, 0.1, 1, 5, 10, or 100 mmol/L for 5 h at room temperature. The supernatant was obtained after 5 h, and the sorption efficiency was estimated as aforementioned.

### EXAFS measurements

EXAFS measurements were performed at the Sr *K*-edge on the BL14B1 line of SPring-8^42, 43^, where X-rays (~16,100 eV) were continuously generated from a bending magnet source. The incident X-rays were monochromatized by two silicon (311) crystals. For the measurements, powder samples were pressed into pellets and placed in the X-ray path. The absorption data were analyzed using the ATENA software package^44^.

## Electronic supplementary material

Calcium-deficient hydroxyapatite as a potential sorbent for strontium
