# Supplementary material for: Calcium-deficient Hydroxyapatite as a Potential Sorbent for Strontium
Source: Sci Rep. 2017 May 18;7:2064. doi: 10.1038/s41598-017-02269-z (PMC5437100; doi:10.1038/s41598-017-02269-z)
Supplement: Supplementary file 1 — Calcium-deficient hydroxyapatite as a potential sorbent for strontium [file 41598_2017_2269_MOESM1_ESM.pdf]

Supplementary Information:

Calcium-deficient hydroxyapatite as a potential sorbent for strontium

Yurina Sekine[1*], Ryuhei Motokawa[1], Naofumi Kozai[2], Toshihiko Ohnuki,[2,3] Daiju Matsumura[1], Takuya Tsuji[1], Riku Kawasaki,[4] and Kazunari Akiyoshi[4,54]

[1]Materials Sciences Research Center, Japan Atomic Energy Agency, 2-4 Shirakata-Shirane, Naka-gun, Tokai, Irabaki, 319-1195, Japan.

[2]Advanced Science Research Center, Japan Atomic Energy Agency, 2-4 Shirakata-Shirane, Naka-gun, Tokai, Ibaraki, 319-1195, Japan.

[3]Laboratory for Advanced Nuclear Energy, Institute of Innovative Research, Tokyo Institute of Technology, Ookayama 2-12-1, Meguro, Tokyo, 152-8550, Japan.

[4]Department of Polymer Chemistry, Graduate School of Engineering, Kyoto University, Katsura, Nishikyo-ku, Kyoto 615-8510, Japan.

[5]ERATO Bio-nanotransporter Project, Japan Science and Technology Agency, Kyoto University, Kastura, Nishikyo-ku, Kyoto 615-8510, Japan.

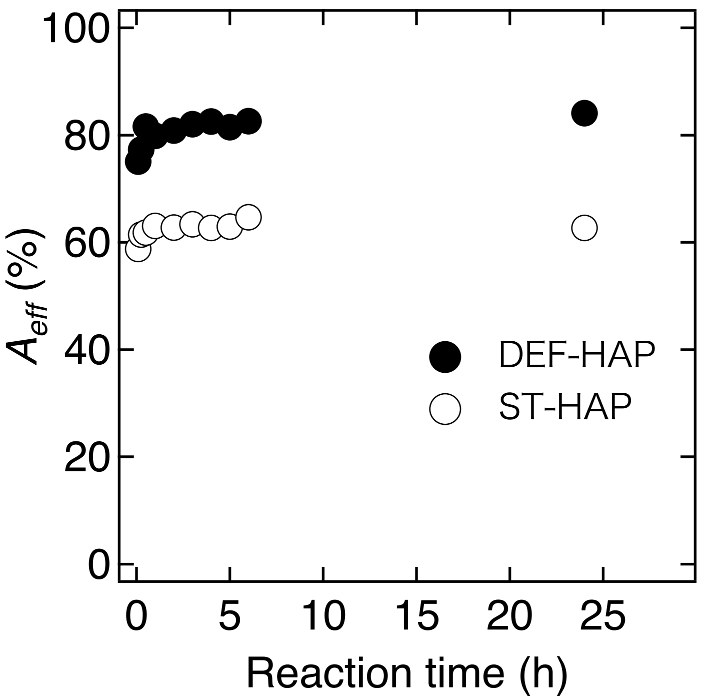

Figure S1: Dependence of reaction time on A_{eff} for DEF-HAP (closed circles) and ST-HAP (open circles). We conducted sorption tests on solution (70 mL) initially containing HAPs (350 mg) and Sr^{2+} (0.1 mmol/L). An aliquots (1 mL) of the suspension was taken at appropriate time intervals. The supernatant was obtained by centrifugation and filtration, and the concentration of Sr^{2+} was determined using ICP-MS. The result showed that the A_{eff} for DEF-HAP and ST-HAP reaches to an equilibrium after 1 h, so 5 h is enough to evaluate the adsorption behavior.

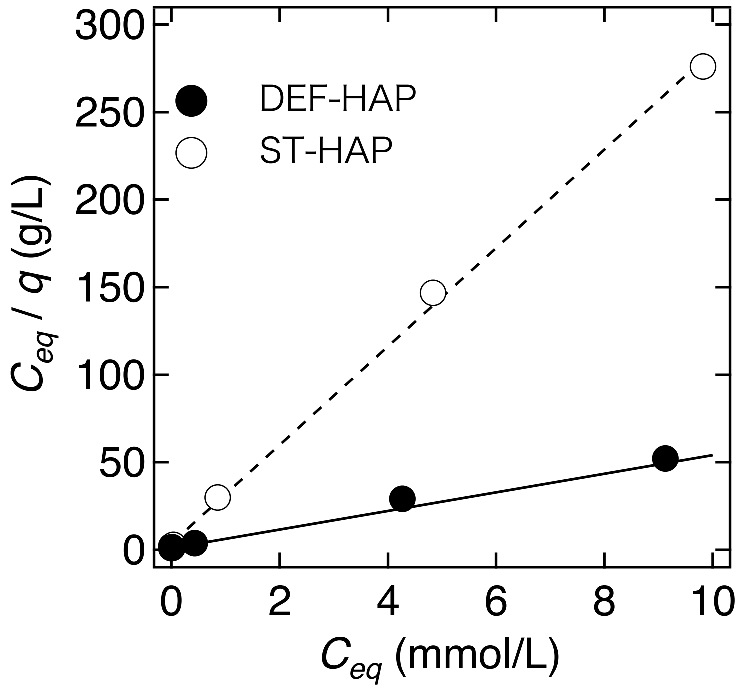

Figure S2: Langmuir isotherm plots for sorption of Sr^{2+} onto DEF-HAP (closed circles) and ST-HAP (open circles). From the obtained values of the slope and the intercept by the fitting, Langmuir isotherm constants, q_{max} and b were estimated.
